# Gastrointestinal tract metastasis with subsequent intussusception and obstruction from an invasive lobular breast cancer: a case report

**DOI:** 10.1093/jscr/rjac623

**Published:** 2023-01-13

**Authors:** Binit Katuwal, Donald Morin, Ramachandra Kolachalam

**Affiliations:** Ascension Providence Hospital, Michigan State University College of Human Medicine, Southfield, MI 49503, USA; Ascension Providence Hospital, Michigan State University College of Human Medicine, Southfield, MI 49503, USA; Ascension Providence Hospital, Michigan State University College of Human Medicine, Southfield, MI 49503, USA

## Abstract

Breast cancer is the commonest cancer in female population with lobular subtype comprising about 10% of all breast cancers. Breast cancer metastasis occurs in 0.3–18% of patients, with lobular cancer again being the most common subtype. We present an 85-year-old female with previous history of right breast lobular carcinoma in situ (LCIS), who was diagnosed to have lobular carcinoma of breast metastasising to stomach after 10 years of initial diagnosis. After 2 years, the patient was found to have metastasis to the terminal ileum and caecum causing intussusception, which led to obstruction. The patient’s primary LCIS was estrogen receptor (ER) positive, progesterone receptor (PR) negative and Her2neu negative, which correlated with both the gastric and ileocecal lesions. The gastric and ileocecal masses both were positive for CK7 and GATA 3 and negative for E-cadherin and CD20. Detailed morphological and immunohistochemical analysis can differentiate primary lobular cancer of the gastrointestinal tract from metastatic cancer.

## INTRODUCTION

Breast cancer is the commonest cancer in female population, with about 29% of all newly diagnosed cancers [1]. It is also the leading cause of all-cancer related deaths for women of age 20–59 years [1]. Invasive lobular cancer comprises about 10% of all breast cancer subtypes, second only to invasive ductal cancer [2]. Almost 6% of all breast cancers metastasise distantly [3]. Metastasis usually occurs in lung liver, brain and bones [4].

Breast cancer metastasis to the gastrointestinal (GI) tract is very uncommon, occurring in about 0.3–18% of patients [5]. It is even rarer to have the gastrointestinal tract as the only site of metastasis. Published data have shown that lobular carcinoma is the histopathological subtype, which is most closely linked to GI tract metastasis [6].

The most common gastrointestinal metastasis site is stomach with about 60% of cases, followed by esophagus, colon, small intestine and rectum [6]. In one literature review, the median survival rate was 12 months from the diagnosis of intestinal metastasis of breast cancer [7]. The GI tract metastasis is usually detected at around 50–78 months after the detection of primary breast cancer [4]. In one instance, gastric metastasis occurred 30 years after mastectomy for breast cancer. Life expectancy appears to increase as the latency period increases [8].

Here we describe a case of an 85-year-old female patient who had a history of left breast cancer and was found to have a gastric and intestinal metastasis.

## CASE PRESENTATION

The patient is an 85-year-old female who had a history of right breast lobular carcinoma in situ (LCIS) diagnosed in 2005. At that time, she opted for treatment with Tamoxifen, which she continued for 4 years. It was stopped due to development of a pulmonary embolism as a result of the treatment.

**Figure 1:**
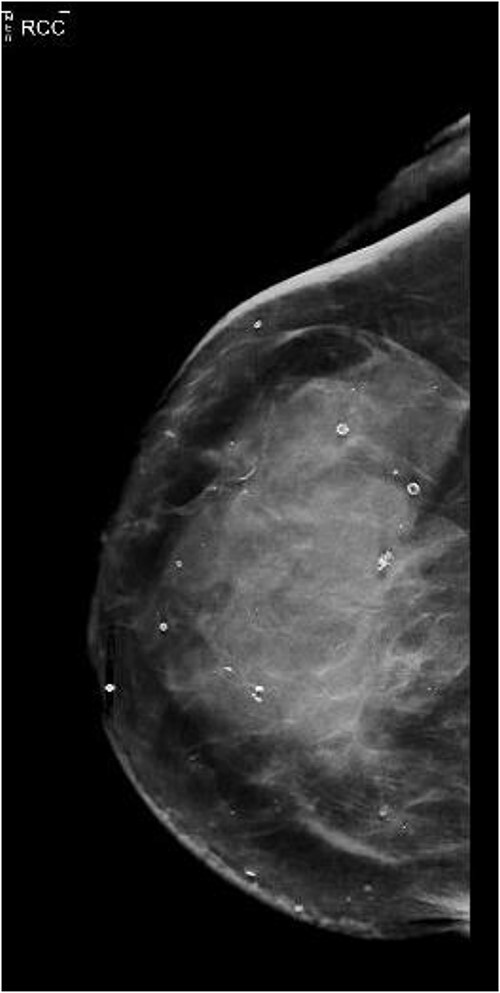
Diagnostic mammography of right breast, craniocaudal (CC).

She presented again in 2019 after a fall when she had also complained of bruising in her right breast. The patient had also complained of occasional nausea and abdominal cramping for the past few weeks. A bilateral mammogram was performed on 22 March 2019, which showed dense skin thickening along with medial inferior asymmetry (Fig. 1). US breast was obtained, and a core needle biopsy was done, which showed invasive lobular carcinoma, T3N1M1, estrogen receptor (ER) positive, progesterone receptor (PR) negative, HER2neu negative.

**Figure 2:**
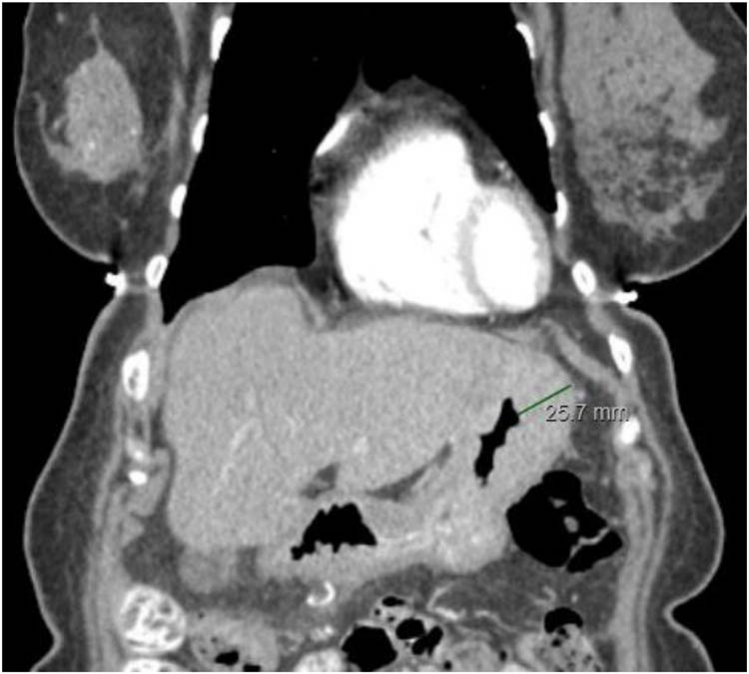
CT abdomen/pelvis displaying gastric wall thickening, coronal view.

The staging work-up was done based upon the national comprehensive cancer network (NCCN) guidelines. A Computed tomography (CT) scan of the abdomen and pelvis was done which showed minimal ascites. There was also evidence of prominence of the stomach wall near the fundus, with thickness measuring 2.2 cm in the CT scan (Fig. 2). This led to an endoscopic gastroduodenoscopy (EGD) that showed a mass in the fundus. Biopsy of the mass revealed lobular carcinoma of the stomach. Bone scan and magnetic resonance imaging (MRI) brain was negative. There was no liver metastasis.

A multidisciplinary conference was done, and after that, the patient was put on Letrozole. Targeted therapy with Ribociclib was started which was discontinued after 5 months due to renal impairment, when the drug was switched to Palbociclib.

On further follow-up, there was regression of breast mass and GI symptoms. CT head, MRI and bone scan was done in 2021, which showed no metastasis.

**Figure 3:**
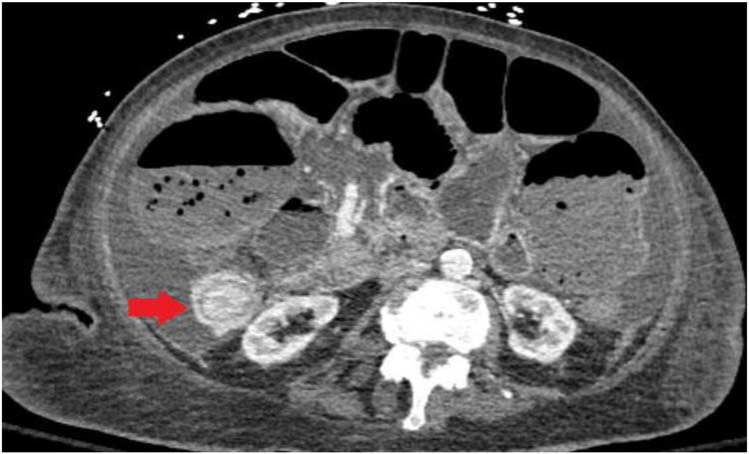
CT abdomen/pelvis displaying intussusception of terminal ileum.

Unfortunately, the patient was diagnosed in April 2021 with complaints of nausea and vomiting and subsequently was found to have an intestine lesion causing intussusception and obstruction, which was perforated, resulting in an emergent surgical exploration and a palliative ileostomy (Fig. 3).

## DISCUSSION

**Figure 4:**
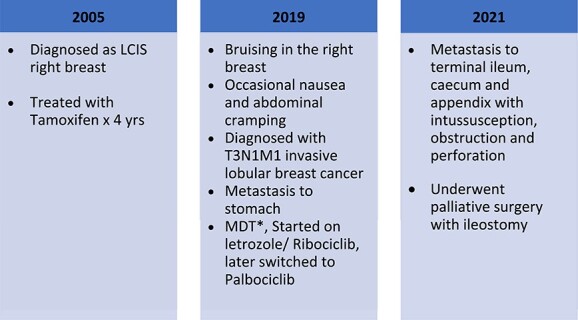
Timeline of the patient presentation and treatment.

In the case described above, there was a diagnosis of LCIS of the right breast in 2005. In 2019, the patient was found to have invasive lobular breast cancer T3N1M1, ER positive, PR positive, HER2neu negative. The patient might have a harbinger of carcinoma at the time of LCIS diagnosis that might have been missed, or there was a subsequent development of carcinoma in a later stage, which is very difficult to ascertain as the patient lost to the regular follow-up (Fig. 4).

This patient’s breast biopsy had shown invasive lobular carcinoma, ER positive (51–100%), PR negative, HER2neu negative.

Subsequent analysis of the gastric biopsy specimen showed invasive lobular carcinoma with ER positive, PR negative, HER2neu negative. Immunostains were positive for DK7 and GATA 3.

The intestinal biopsy during the exploratory laparotomy showed metastatic lobular carcinoma involving terminal ileum, colon and appendix, ER positive, PR negative, HER2neu negative. Immunostains were positive for CK7, GATA 3. CD20 was negative, and E-cadherin stain was lost in tumor cells (Table 1). 

**Table 1:** Immunostaining profile comparison of tumor cells in primary vs metastasis sites

**IHC Marker**	**Breast tumor cells**	**Gastric metastasis**	**Colonic metastasis**
ER	Positive	Positive	Positive
PR	Negative	Negative	Negative
Her2neu	Negative	Negative	Negative
E-cadherin	NA	Negative	Negative
CK7	NA	Positive	Positive
GATA 3	NA	Positive	Positive
CD20	NA	Negative	Negative

In case of invasive lobular carcinoma, diagnosis of gastric metastasis is challenging as it might mimic primary cancer [5]. Invasive lobular carcinoma of the breast is the cause of gastric metastasis from breast in about 97% of the cases. Histopathological analysis can be helpful in distinguishing between these two forms of cancers. The first important difference is the localisation of tumor cells: mucosa is generally involved in gastric cancer, whereas submucosal layer is usually affected in metastatic disease [9].

Both the cancer forms can have signet rings, but gastric signet rings have multivacuolated cytoplasm, whereas the lobular metastatic cells have a well-defined uni-vacuolated cytoplasm [10]. During endoscopy, gastric metastasis of LCIS tends to reveal a diffuse linitis plastica-like infiltration, as opposed to a discrete mass seen in primary gastric neoplasms [5].

A detailed immunohistopathological analysis can finally help in distinguishing two entities with significant specificity. ER, PR, GCDFP are highly specific to metastatic breast cancer. CK20, DAS-1, MUC2, MUC5AC, MUC6, CDX2 are specific to primary gastric carcinomas [10].

## CONCLUSION

Gastric metastasis and subsequent colonic metastasis are very rare forms of breast cancer. Invasive lobular cancer is the most common form of breast cancer that is associated with GI tract metastasis. It is important to have an index of suspicion if the patient is diagnosed with GI symptoms and has a previous history of lobular breast cancer, as these patients might have a metastasis. A detailed morphological and immunohistochemical analysis can aid in differentiating primary gastric cancer from a gastric metastasis arising from an invasive lobular breast cancer.

## CONFLICT OF INTEREST STATEMENT

None declared.

## FUNDING

None.
